# Development of an IL-6- and Th1/Treg-based nomogram for identifying low bone mass in patients with rheumatoid arthritis

**DOI:** 10.3389/fimmu.2026.1952321

**Published:** 2026-10-21

**Authors:** Zemin Liu, Yueqi Li, Dong Wang, Chaoqi Wang, Ze Zhang, Chao Li, Xiaoli Li, Yonghong Zhang, Yan Li

**Affiliations:** 1 Department of Orthopaedics, Second Hospital of Shanxi Medical University, Taiyuan, Shanxi, China; 2 Graduate School, Shanxi Medical University, Taiyuan, Shanxi, China; 3 Department of Infectious Diseases, Fenyang Hospital of Shanxi Province (First Hospital of Shanxi University of Medicine), Fenyang, Shanxi, China; 4 School of Basic Medicine, Shanxi Medical University, Taiyuan, Shanxi, China

**Keywords:** IL-6, low bone mass, nomogram, rheumatoid arthritis, Th1/Treg

## Abstract

**Objective:**

This study aimed to analyze clinical and immunological characteristics in patients with rheumatoid arthritis (RA) according to bone loss status, integrate traditional risk factors with immune-inflammatory indicators, and construct and validate an individualized nomogram for estimating the probability of concurrent BMD-defined low bone mass in patients with RA.

**Methods:**

A total of 442 patients with RA were enrolled and divided into normal bone mass (RA-N, n=219) and low bone mass (RA-LBM, n=223) groups based on bone mineral density values. Data on the clinical characteristics, laboratory parameters, peripheral blood lymphocyte subsets, and cytokine levels were collected for both groups. The Benjamini–Hochberg (BH) method was applied for multiple testing correction. Univariate logistic regression was used to screen relevant variables, and the random forest algorithm (mean decrease Gini importance ranking and out-of-bag error analysis) was employed to determine the optimal combination of variables. Multivariate logistic regression was performed to identify factors independently associated with low bone mass, based on which a nomogram for identifying concurrent low bone mass was constructed. The discriminative ability of the model was evaluated using the receiver operating characteristic curve, calibration was assessed using the calibration curve and Hosmer–Lemeshow test, and clinical net benefit was evaluated using decision curve analysis.

**Results:**

Patients in the RA-LBM group exhibited significantly higher values for age, disease duration, erythrocyte sedimentation rate, aspartate aminotransferase/alanine aminotransferase ratio, and alkaline phosphatase, while body mass index (BMI), red blood cell count, hemoglobin levels, albumin levels, and albumin/globulin ratio were significantly lower than those in the RA-N group (BH-adjusted P<0.05). Immunological analysis revealed that interleukin-6 (IL-6) levels were significantly elevated in the RA-LBM group (BH-adjusted P<0.001), and the T helper 1 cell/regulatory T cell (Th1/Treg) ratio was also significantly increased (BH-adjusted P = 0.016). Random forest analysis followed by multivariate logistic regression ultimately identified age, Th1/Treg ratio, BMI, IL-6, albumin, and uric acid levels as factors independently associated with low bone mass in patients with RA (BH-adjusted P<0.05). Model 1 constructed based on these six variables achieved an area under the curve (AUC) of 0.906 (95% CI: 0.877–0.935), which was significantly higher than that of model 2 containing only traditional risk factors (AUC = 0.861, P = 0.001). The AUCs of model 1 in the training and validation sets were 0.906 and 0.905, respectively. The Hosmer–Lemeshow test indicated good model calibration. Decision curve analysis showed that the model provided a higher net benefit than the treat-all and treat-none strategies across a range of threshold probabilities.

**Conclusion:**

This study integrates immune-inflammatory indicators, such as IL-6 and Th1/Treg ratio, with traditional risk factors to construct a nomogram for identifying concurrent BMD-defined low bone mass in patients with RA. The model showed favorable discrimination and calibration and may provide a useful tool for estimating the probability of concurrent low bone mass in this population.

## 1 Introduction

Rheumatoid arthritis (RA) is a common systemic autoimmune disease characterized by persistent synovial inflammation, pannus formation, and cartilage and bone destruction, ultimately leading to joint deformities and functional loss along with systemic bone mass loss and various extra-articular manifestations (1, 2). The global prevalence of RA ranges from 0.2% to 1%, with a female-to-male ratio of 2–3:1; both prevalence and incidence have shown an increasing trend annually (3, 4). Patients with RA exhibit elevated levels of local and systemic inflammation that promote bone loss by enhancing bone resorption and suppressing bone formation (5). Osteoporosis is a systemic skeletal disease characterized by a disrupted bone microarchitecture and reduced bone strength resulting from diminished bone formation and/or enhanced bone resorption. It is a common extra-articular complication in patients with RA, with a prevalence of 27.6%, approximately twice that in the general population (6). The fracture risk in patients with RA is 40% higher than that in non-RA populations (7). Approximately one-third of RA-related deaths in the United States are attributed to osteoporotic fractures (6). RA is an independent risk factor for osteoporosis and fractures (8, 9). Therefore, identifying patients with RA who have low bone mass and characterizing factors associated with this condition are clinically important.

Although bone loss in patients with RA may be associated with multiple factors, including chronic inflammation, reduced physical activity, metabolic disorders, and glucocorticoid use (5), these factors alone do not fully account for the high prevalence of osteoporosis in this population. Takuya et al. (10) reported in a clinical study that RA demonstrated the strongest association with osteoporosis among all evaluated risk factors. A Mendelian randomization study based on an East Asian population found that RA remained positively correlated with osteoporosis, even after adjusting for confounding factors (11).

The factors associated with osteoporosis in patients with RA have not been fully elucidated. In recent years, osteoimmunology research has revealed that patients with RA exhibit immune dysregulation characterized by increased Th17 cells, an altered Th17/Treg balance, and aberrant B-cell activation, together with elevated levels of pro-inflammatory cytokines such as IL-6, IL-17, and TNF-α. These immune abnormalities not only contribute to synovial inflammation but are also involved in osteoclast activation through the RANKL-OPG axis and non-RANKL-dependent immune pathways (12–14). Multiple studies have quantitatively linked these immune indicators to decreased bone mineral density (BMD) and increased fracture risk (15–17). These findings suggest that immune cells and inflammatory cytokines may provide additional information for identifying patients with RA with low bone mass.

Current evidence has demonstrated correlations between immune-inflammatory status, RA, and an increased risk of bone loss. However, immune-inflammatory indicators have not yet been adequately incorporated into multivariable models for identifying low bone mass in patients with RA. Previous studies have predominantly focused on conventional clinical risk factors and bone metabolic markers. Conventional clinical risk factors include advanced age, female sex, postmenopausal status, low body mass index, smoking, longer RA duration, high disease activity, functional disability, and glucocorticoid exposure (18, 19). Bone metabolic markers mainly include bone resorption markers, such as C-terminal telopeptide of type I collagen and N-terminal telopeptide of type I collagen, and bone formation markers, such as procollagen type I N-terminal propeptide, osteocalcin, and bone-specific alkaline phosphatase (20). Although these conventional markers are valuable for assessing bone health, they do not directly reflect the immune-inflammatory abnormalities associated with bone loss in RA. Recently, Zhang et al. (21) reported an association between an elevated neutrophil percentage-to-serum albumin ratio and bone loss in patients with RA. Qiu et al. (22) identified associations between immunomodulatory factors, including IL-17, IL-6, TNF-α, IL-10, and IL-4, and osteoporosis in patients with RA. These findings provide a rationale for further evaluating whether immune-inflammatory markers provide additional information beyond conventional factors for identifying low bone mass in patients with RA.

Nomogram models provide a visual and clinically interpretable approach for estimating the probability of a concurrent clinical condition. In this study, we retrospectively analyzed clinical data from patients with RA and developed and internally validated a novel nomogram integrating demographic characteristics, serum immune cells, inflammatory cytokines, and metabolic indicators. The model was designed to estimate the probability of concurrent BMD-defined low bone mass and to evaluate whether immune-inflammatory markers provide additional discriminatory information beyond conventional factors.

## 2 Materials and methods

### 2.1 Study population

This retrospective study consecutively screened patients with RA who were hospitalized in the Department of Rheumatology and Immunology, Second Hospital of Shanxi Medical University, between January 1, 2018, and January 1, 2025, according to the predefined inclusion and exclusion criteria. The primary reason for hospitalization was worsening of RA-related disease activity or symptoms requiring further clinical evaluation and management. For patients with multiple hospitalizations during the study period, only data from the first eligible hospitalization were included, and subsequent hospitalizations were excluded to avoid repeated observations from the same individual. This study was approved by the Ethics Committee of the Second Hospital of Shanxi Medical University (approval number 2025-YX-326). The inclusion criteria were as follows: diagnosis fulfilling the 2010 American College of Rheumatology/European League Against Rheumatism (ACR/EULAR) classification criteria for RA (23) and age >18 years.

A total of 697 hospitalized patients with RA were screened during the study period. After applying the predefined inclusion and exclusion criteria, 442 patients were included in the final analysis and classified according to BMD status into the RA-LBM group (n = 223) and the RA-N group (n = 219). Of the 255 excluded patients, 103 were excluded because of missing BMD assessment, 124 because of coexisting autoimmune or inflammatory rheumatic diseases, and 28 because of infectious diseases. Among the 124 patients with coexisting autoimmune or inflammatory rheumatic diseases, 16 had primary Sjögren syndrome, 73 had secondary Sjögren syndrome, 14 had ankylosing spondylitis, 9 had systemic lupus erythematosus, and 12 had gout (**Supplementary Figure 1**). Among the 442 included patients, 296 (66.97%) underwent BMD assessment at both the femoral neck and lumbar spine, 77 (17.42%) had femoral neck measurements only, and 69 (15.61%) had lumbar spine measurements only. For patients with measurements at both sites, the lower T-score of the femoral neck and lumbar spine was used for classification. Patients with a T-score < −1.0 at either site were classified into the BMD-defined low bone mass group (RA-LBM), whereas those with T-scores ≥ −1.0 at both sites were classified into the normal bone mass group (RA-N) (24). For patients with measurements available at only one site, classification was based on the T-score at the available site.

### 2.2 Data collection

Demographic, clinical, and laboratory data, including age, sex, disease duration, body mass index (BMI), smoking history, alcohol consumption history, hypertension, diabetes mellitus, medications for RA treatment (including NSAIDs, glucocorticoids, conventional synthetic DMARDs, biologic DMARDs, and JAK inhibitors), medications for bone loss prevention or treatment, complete blood count, hepatic and renal function tests, lipid profile, erythrocyte sedimentation rate (ESR), C-reactive protein, immunoglobulins, peripheral blood lymphocyte subset counts and percentages, and inflammatory cytokine levels, were collected from the hospital’s electronic medical record system. Medication exposure was assessed at admission, while biomarker measurements and BMD assessments were performed during the same hospitalization. Trained personnel independently performed data collection, entry, and verification. If a parameter was measured multiple times after admission, the results of the first measurement were recorded.

### 2.3 Peripheral blood lymphocyte subset analysis protocol

Venous blood samples were collected from each participant in heparin-coated tubes to preserve cellular integrity. Peripheral blood mononuclear cells were isolated using Ficoll-Hypaque density gradient centrifugation, resuspended at a concentration of 1–2 × 10^6^ cells/mL, and stained with fluorochrome-conjugated monoclonal antibodies. For T lymphocyte analysis, cells were stained with anti-CD3-FITC, anti-CD8-PE, anti-CD45-PerCP, and anti-CD4-APC; for B and NK cell analysis, anti-CD3-FITC, anti-CD16/CD56-PE, anti-CD45-PerCP, and anti-CD19-APC were used. Th1 cells were identified using anti-CD4-FITC combined with anti-IFN-γ-APC, Th2 cells with anti-CD4-FITC and anti-IL-4-PE, and Th17 cells with anti-CD4-FITC and anti-IL-17A-PE. For Treg cell detection, the cells were stained with anti-CD4-FITC, anti-CD25-APC, and anti-FOXP3-PE. Prior to intracellular cytokine staining for Th1/Th2/Th17 panels, cells were stimulated with phorbol 12-myristate 13-acetate and ionomycin for 4–6 h, followed by fixation and permeabilization. The Th1/Treg ratio was calculated using the absolute counts of Th1 and Treg cells. Because intracellular cytokine staining was performed after PMA/ionomycin stimulation, the resulting Th1 measurements reflect inducible cytokine-producing capacity under ex vivo stimulation rather than the unstimulated *in vivo* Th1 state. All antibodies were purchased from BD Biosciences (San Jose, CA, USA) and used according to the manufacturer’s instructions. Lymphocyte subset counts (absolute and relative) were quantified using a BD FACSCanto™ flow cytometer (BD Biosciences, San Jose, CA, USA) with BD Multitest™ software.

### 2.4 Serum cytokine quantification via multiplex bead assay

Serum cytokine measurements were performed by the hospital clinical laboratory during each patient’s hospitalization as part of routine laboratory testing, and the reported results were retrospectively extracted from the electronic medical records for the present study. Information regarding sample processing, assay procedures, and reagents was verified with the clinical laboratory. Following the collection of peripheral blood samples from patients, serum was isolated within 1 h. The isolation procedure entailed an initial centrifugation (4 °C for 10 min at 800×*g*), followed by centrifugation at 2000×*g* for 15 min to eliminate cellular remnants. Serum levels of IL-2, IL-4, IL-6, IL-10, IL-17, IFN-γ, and TNF-α were quantified using a cytometric bead array (CBA) kit (Jiangxi Cellgene Biotechnology Co., Ltd., Jiangxi, China) according to the manufacturer’s instructions. Acquisition occurred on the BD FACSCanto™ flow cytometer (BD Biosciences, San Jose, CA, USA), with resultant quantitative metrics processed through BD FCAP Array™ v3.0 software.

### 2.5 Statistical analysis

Statistical analyses were performed using GraphPad Prism 10 (San Diego, CA, USA) and R software (version 4.4.2; USA). The normality of continuous variables was assessed using the Shapiro–Wilk test. Normally distributed data are presented as mean ± standard deviation, and intergroup comparisons were conducted using the independent sample t-test. Non-normally distributed data are presented as median (interquartile range), and intergroup comparisons were performed using the Mann–Whitney U test. Categorical variables are expressed as frequencies and percentages, and intergroup comparisons were analyzed using the chi-square test. Statistical significance was set at P <0.05.

To control for false positives resulting from multiple comparisons, all P-values were adjusted for the false discovery rate using the Benjamini–Hochberg (BH) method. Differences were considered statistically significant at a BH-adjusted P-value <0.05. Patients were randomly divided into training and validation sets at an 8:2 ratio using outcome-stratified sampling with a fixed random seed (123) to ensure reproducibility. All variable-selection and model-development procedures were performed exclusively in the training set. Missing data were imputed using the mice package in R. Predictive mean matching was employed for continuous variables, and logistic regression was used for binary variables. The number of imputations was set to 5 (m=5) with 10 iterations per imputation (maxit=10). Imputation was performed separately for the training and test sets. Outcome variables were not used during the imputation of the test set to avoid information leakage. The results from the imputed datasets were pooled using Rubin’s rules.

Following the analytical framework used in previous studies (25, 26), univariable logistic regression was initially performed on all candidate predictors, and variables associated with low bone mass in patients with RA (P<0.05) were subsequently included in the random forest analysis. Subsequently, the random forest algorithm was employed to evaluate the importance of the screened variables using the mean decrease Gini coefficient and out-of-bag (OOB) error analysis to determine the optimal number of variables. Variables selected by random forest underwent multivariate logistic regression analysis to identify independent associated with low bone mass in RA, based on which a nomogram for estimating the probability of concurrent low bone mass was constructed. Model performance was evaluated as follows: area under the receiver operating characteristic curve (AUC) for discriminative ability, calibration curve and Hosmer–Lemeshow goodness-of-fit test for calibration, and decision curve analysis for clinical net benefit.

## 3 Results

### 3.1 Comparison of demographic, clinical, and laboratory data

The demographic, clinical, and laboratory data of the two groups are presented in **Table 1**. Patients in the RA-LBM group were significantly older than those in the RA-N group (63 [55, 72] vs. 51 [40, 58] years, BH-adjusted P<0.001), and the RA-LBM group had a higher proportion of female patients (193 [86.55%] vs. 173 [79.00%], unadjusted P = 0.035, BH-adjusted P = 0.116). The BMI of patients in the RA-LBM group was lower than that of patients in the RA-N group (21.88 [19.17, 24.08] vs. 23.44 [21.23, 25.41 kg/m^2^], BH-adjusted P<0.001). Notably, disease duration in the RA-LBM group was significantly longer than that in the RA-N group (120 [36, 222] vs. 36 [12, 114], BH-adjusted P<0.001). A subset of patients in both groups received pharmacological treatment, with the main drug classes including nonsteroidal anti-inflammatory drugs, glucocorticoids, conventional synthetic disease-modifying antirheumatic drugs (csDMARDs), biologic DMARDs (bDMARDs), Janus kinase (JAK) inhibitors, and calcium supplements. Among these, the proportion of patients taking csDMARDs was higher in the RA-LBM group (134 [60.09%] vs. 111 [50.68%]; unadjusted P = 0.047, BH-adjusted P = 0.131). Comparisons of laboratory parameters revealed that ESR, the aspartate aminotransferase/alanine aminotransferase (AST/ALT) ratio, and alkaline phosphatase levels were higher in the RA-LBM group than in the RA-N group, and these differences remained statistically significant after BH adjustment (P<0.05). Red blood cell count (RBC), hemoglobin levels, albumin levels, and albumin/globulin ratio were lower in the RA-LBM group than in the RA-N group, and these differences also remained significant after correction (P<0.05). Additionally, GLO, uric acid, TSH, and IgA levels were higher, whereas ALT levels were lower in the RA-LBM group than in the RA-N group; however, none of these differences reached statistical significance after adjustment (P≥0.05).

**Table 1:** Demographic, clinical, and laboratory characteristics of patients in the RA-LBM and RA-N groups.

Characteristics	RA-LBM (n=223)	RA-N (n=219)	t/z/χ^2^	Unadjusted p-value	BH-adjusted p-value
Demographics					
Male (%)	30 (13.45)	46 (21.00)	6.343	0.012*	0.058
Female (%)	193 (86.55)	173 (79.00)	4.425	0.035*	0.116
Age (years)	63.00 (55.00, 72.00)	51.00 (40.00, 58.00)	10.334	<0.001***	<0.001***
BMI (Kg/m^2^)	21.88 (19.17, 24.08)	23.44 (21.23, 25.41)	-4.101	<0.001***	<0.001***
Course of disease (month)	120.00 (36.00, 222.00)	36.00 (12.00, 114.00)	6.007	<0.001***	<0.001***
Smoke (%)	24 (10.76)	25 (11.42)	0.048	0.827	0.913
Drink (%)	13 (5.83)	12 (5.48)	0.025	0.873	0.925
Diabetes (%)	15 (6.73)	7 (3.20)	2.911	0.088	0.203
Hypertension (%)	60 (26.91)	48 (21.92)	1.489	0.222	0.359
Current use of medication					
NSAIDs (%)	107 (47.98)	93 (42.47)	1.357	0.244	0.359
GC (%)	95 (42.60)	83 (37.90)	1.015	0.314	0.427
csDMARDs (%)	134 (60.09)	111 (50.68)	3.956	0.047*	0.131
bDMARDs (%)	19 (8.52)	11 (5.02)	2.136	0.144	0.285
JAK inhibitors (%)	10 (4.48)	9 (4.11)	0.038	0.846	0.915
Calcium supplement (%)	23 (10.31)	21 (9.59)	0.065	0.799	0.901
Laboratory characteristics					
CRP (mg/L)	12.71 (3.74, 38.76)	8.17 (3.33, 29.86)	1.670	0.083	0.200
ESR (mm/h)	61.20 (28.20, 99.80)	39.20 (17.80, 77.60)	4.452	<0.001***	<0.001***
WBC (*10^9^/L)	6.11 (4.67, 8.16)	5.76 (4.41, 7.34)	1.392	0.159	0.292
RBC (*10^12^/L)	4.05 (3.71, 4.37)	4.21 (3.93, 4.57)	-3.589	<0.001***	0.003**
HGB (g/L)	117.80 (103.40, 128.20)	123.80 (110.70, 134.00)	-3.145	0.002**	0.015*
PLT (*10^9^/L)	270.60 (215.00, 336.40)	252.00 (194.70, 318.30)	1.889	0.074	0.187
LY (*10^9^/L)	1.42 (1.04, 1.87)	1.38 (1.07, 1.89)	-0.022	0.944	0.944
LY%	23.66 (17.44, 31.26)	24.58 (19.24, 32.10)	-1.108	0.228	0.359
MONO (*10^9^/L)	0.40 (0.29, 0.58)	0.43 (0.31, 0.55)	-0.344	0.760	0.876
MONO%	6.80 (5.37, 8.43)	7.00 (5.40, 8.97)	-1.263	0.231	0.359
NEUT (*10^9^/L)	4.07 (2.70, 5.55)	3.79 (2.76, 5.28)	0.976	0.347	0.449
NEUT%	67.28 (58.74, 74.69)	66.52 (56.77, 72.84)	1.112	0.281	0.403
ALT (U/L)	14.90 (10.76, 24.56)	16.76 (11.77, 30.62)	-2.277	0.023*	0.081
AST (U/L)	19.84 (16.01, 26.47)	20.52 (16.60, 27.06)	-1.191	0.230	0.359
AST/ALT	1.32 (1.03, 1.62)	1.23 (0.89, 1.55)	2.634	0.007**	0.041*
TBIL (μmol/L)	9.86 (7.51, 12.48)	9.92 (8.21, 12.87)	-1.472	0.145	0.285
DBIL (μmol/L)	1.90 (1.40, 2.50)	1.90 (1.58, 2.52)	-0.782	0.322	0.427
IBIL (μmol/L)	7.95 (6.20, 9.97)	8.12 (6.57, 10.82)	-1.261	0.241	0.359
TP (g/L)	70.28 (65.99, 75.62)	71.28 (66.60, 76.73)	-0.704	0.486	0.599
ALB (g/L)	34.86 (31.36, 38.70)	38.12 (34.72, 41.39)	-5.923	<0.001***	<0.001***
GLO (g/L)	34.76 (29.69, 40.21)	33.04 (28.42, 38.32)	2.282	0.017*	0.069
A/G	1.04 (0.84, 1.26)	1.11 (0.96, 1.35)	-2.999	0.003**	0.020*
ALP (U/L)	91.80 (75.80, 113.40)	85.80 (67.60, 107.40)	2.644	0.008**	0.042*
GGT (U/L)	22.70 (15.85, 40.52)	22.40 (15.19, 41.30)	0.318	0.890	0.925
UREA (mmol/L)	5.06 (4.20, 6.26)	5.06 (4.10, 6.19)	-0.195	0.939	0.944
Cr (μmol/L)	52.00 (45.00, 59.00)	53.20 (47.00, 60.00)	-1.586	0.108	0.239
Uric (μmol/L)	287.80 (225.00, 387.10)	271.60 (224.30, 339.50)	2.162	0.038*	0.118
CHO (mmol/L)	4.16 (3.56, 4.76)	4.18 (3.64, 4.85)	-0.784	0.497	0.599
TG (mmol/L)	1.08 (0.84, 1.43)	1.12 (0.84, 1.49)	-0.834	0.301	0.420
HDL (mmol/L)	1.20 (0.99, 1.43)	1.17 (0.97, 1.39)	0.864	0.496	0.599
LDL (mmol/L)	2.31 (1.84, 2.78)	2.35 (1.91, 2.90)	-1.307	0.160	0.292
TSH (mIU/L)	2.76 (1.39, 4.90)	2.08 (1.24, 3.83)	2.244	0.016*	0.069
FT4 (pmol/L)	11.70 (10.26, 13.51)	11.17 (9.96, 12.92)	1.942	0.070	0.186
FT3 (pmol/L)	4.64 (4.15, 5.10)	4.69 (4.27, 5.17)	-1.311	0.228	0.359
IgA (g/L)	3.28 (2.30, 4.70)	2.86 (2.19, 3.93)	2.314	0.019*	0.072
IgG (g/L)	14.36 (11.21, 19.08)	14.27 (11.10, 18.95)	0.134	0.664	0.782
IgM (g/L)	1.26 (0.90, 1.91)	1.17 (0.90, 1.65)	1.522	0.145	0.285
25-hydroxy vitamin D (ng/mL)	14.19 (7.49, 24.16)	13.19 (7.04, 23.00)	1.054	0.041*	0.121

BMI, body mass index; NSAIDs, nonsteroidal anti-inflammatory drugs; GC, glucocorticoids; csDMARDs, conventional synthetic disease-modifying antirheumatic drugs; bDMARDs, biologic disease-modifying antirheumatic drugs; JAK, Janus kinase; CRP, C-reactive protein; ESR, erythrocyte sedimentation rate; WBC, white blood cell; RBC, red blood cell; HGB, hemoglobin; PLT, platelet; LY, lymphocyte; MONO, monocyte; NEUT, neutrophil; ALT, alanine aminotransferase; AST, aspartate aminotransferase; TBIL, total bilirubin; DBIL, direct bilirubin; IBIL, indirect bilirubin; TP, total protein; ALB, albumin; GLO, globulin; A/G, albumin/globulin ratio; ALP, alkaline phosphatase; GGT, gamma-glutamyl transferase; Cr, creatinine; UA, uric acid; CHOL, cholesterol; TG, triglyceride; HDL, high-density lipoprotein; LDL, low-density lipoprotein; TSH, thyroid-stimulating hormone; FT4, free thyroxine; FT3, free triiodothyronine; IgA, immunoglobulin A; IgG, immunoglobulin G; IgM, immunoglobulin M. *P<0.05, **P<0.01, ***P<0.001.

### 3.2 Comparison of peripheral blood lymphocyte subsets

To investigate the role of immune cells in bone loss in patients with RA, we analyzed the counts and percentages of immune cells in both groups using flow cytometry (**Table 2**). The percentage of NK cells and Th1/Treg ratio were both higher in the RA-LBM group than in the RA-N group, and these differences remained statistically significant after BH correction (P<0.05). Additionally, the NK cell count was higher in the RA-LBM group than in the RA-N group before BH correction (P = 0.038); however, this difference was not statistically significant after adjustment (P = 0.145). The T cell percentage was lower in the RA-LBM group than in the RA-N group before correction; however, this difference was also not significant after adjustment. None of the remaining parameters showed statistically significant differences before or after BH correction. As described in the Methods, the Th1-related measurements were obtained after PMA/ionomycin stimulation and therefore reflect inducible cytokine-producing capacity under ex vivo stimulation rather than unstimulated *in vivo* Th1 activity.

**Table 2:** Immune cell counts and percentages in the RA-LBM and RA-N groups.

(A)	RA-LBM (n=223)	RA-N (n=219)	t/z/χ^2^	Unadjusted p-value	BH-adjusted p-value
Total T (cells/μL)	973.68 (706.06, 1378.85)	1019.63 (778.12, 1380.51)	-0.788	0.417	0.597
Total B (cells/μL)	145.06 (89.54, 237.14)	158.38 (104.46, 242.63)	-1.254	0.210	0.461
NK (cells/μL)	164.04 (102.70, 273.15)	146.15 (89.19, 234.36)	2.005	0.038*	0.145
CD4+ T (cells/μL)	601.81 (411.00, 814.99)	577.74 (431.80, 787.09)	0.221	0.842	0.894
CD8+ T (cells/μL)	343.05 (234.22, 494.83)	360.82 (256.80, 508.37)	-1.127	0.269	0.482
Th1 (cells/μL)	82.24 (54.22, 131.09)	79.56 (47.03, 134.62)	0.801	0.450	0.624
Th2 (cells/μL)	6.38 (4.05, 9.85)	6.24 (3.80, 9.88)	-0.009	0.880	0.911
Th17 (cells/μL)	7.46 (4.24, 11.64)	7.58 (4.69, 11.80)	-0.608	0.693	0.827
Treg (cells/μL)	23.78 (14.74, 34.89)	24.96 (16.57, 37.71)	-0.921	0.312	0.509
(B)	RA-LBM (n=223)	RA-N (n=219)	t/z/χ^2^	Unadjusted P-value	BH-adjusted P-value
T%	72.82 (65.72, 78.15)	74.50 (67.29, 79.45)	-2.044	0.039*	0.145
B%	11.27 (6.90, 16.16)	11.61 (7.88, 16.39)	-0.544	0.482	0.647
NK%	13.20 (8.50, 18.69)	10.24 (7.36, 15.46)	3.078	0.002**	0.016*
CD4+ T%	44.35 (35.72, 49.51)	43.18 (36.70, 48.67)	0.429	0.720	0.832
CD8+ T%	25.80 (21.36, 30.01)	26.77 (21.02, 33.09)	-0.952	0.347	0.534
CD4+ T/CD8+ T	1.65 (1.30, 2.17)	1.60 (1.17, 2.22)	0.409	0.616	0.791
Th1%	15.04 (10.72, 22.00)	14.79 (9.84, 19.63)	1.538	0.110	0.315
Th2%	1.11 (0.76, 1.55)	1.05 (0.80, 1.43)	0.308	0.725	0.832
Th17%	1.30 (0.85, 1.71)	1.36 (0.86, 1.94)	-1.215	0.208	0.461
Treg%	4.01 (3.13, 5.19)	4.17 (3.29, 5.58)	-1.205	0.253	0.462
Th1/Th2	13.21 (8.57, 25.09)	12.80 (9.10, 19.03)	0.917	0.377	0.559
Th17/Treg	0.33 (0.20, 0.47)	0.32 (0.20, 0.47)	-0.197	0.833	0.894
Th1/Treg	4.56 (2.79, 7.50)	3.24 (2.06, 5.00)	4.827	<0.001***	<0.001***
Th2/Treg	0.27 (0.18, 0.39)	0.25 (0.18, 0.37)	1.067	0.406	0.592
B/Treg	5.77 (3.45, 9.84)	6.07 (3.83, 9.75)	-0.553	0.663	0.815
NK/Treg	6.19 (3.88, 11.92)	5.82 (3.35, 11.30)	1.001	0.247	0.461

T, T lymphocyte; B, B lymphocyte; NK, natural killer cell; CD4+ T, CD4-positive T lymphocyte; CD8+ T, CD8-positive T lymphocyte; Th1, T helper 1 cell; Th2, T helper 2 cell; Th17, T helper 17 cell; Treg, regulatory T cell. *P<0.05, **P<0.01, ***P<0.001.

(A) Absolute counts of peripheral immune cell subsets.

(B) Percentages and ratios of peripheral immune cell subsets.

### 3.3 Comparison of peripheral blood cytokine levels

To determine whether common cytokines are involved in the presence of low bone mass in RA, we compared peripheral blood cytokine levels between the two groups (**Table 3**). IL-6 levels were significantly higher in the RA-LBM group than in the RA-N group (30.35 [10.52, 162.47] vs. 12.44 [5.65, 46.87], BH-adjusted P<0.001). No significant differences were observed for the remaining parameters before or after correction.

**Table 3:** Cytokine levels in the RA-LBM and RA-N groups.

Cytokines	RA-LBM (n=223)	RA-N (n=219)	t/z/χ^2^	Unadjusted p-value	BH-adjusted p-value
IL-2 (pg/ml)	2.86 (1.66, 4.35)	2.50 (1.48, 4.10)	1.252	0.280	0.483
sIL-2R (pg/ml)	602.69 (322.22, 1101.72)	638.05 (358.91, 1047.15)	-0.602	0.650	0.815
IL-4 (pg/ml)	2.89 (1.77, 4.65)	2.74 (1.92, 4.49)	0.383	0.706	0.832
IL-6 (pg/ml)	30.35 (10.52, 162.47)	12.44 (5.65, 46.87)	4.964	<0.001***	<0.001***
IL-10 (pg/ml)	5.71 (3.60, 8.09)	5.64 (4.20, 8.26)	-0.907	0.246	0.461
IL-17 (pg/ml)	6.66 (2.48, 18.17)	5.74 (2.70, 14.56)	0.741	0.354	0.534
IFN-γ (pg/ml)	4.00 (2.81, 6.65)	4.09 (2.95, 6.37)	-0.333	0.679	0.822
TNF-α (pg/ml)	4.02 (2.56, 7.73)	3.75 (2.61, 6.64)	0.865	0.433	0.611

IL-2, interleukin-2; IL-4, interleukin-4; IL-6, interleukin-6; IL-10, interleukin-10; IL-17, interleukin-17; IFN-γ, interferon-γ; TNF-α, tumor necrosis factor-α. ***P<0.001.

### 3.4 Development of a nomogram for identifying concurrent low bone mass risk in patients with RA

The 442 patients with RA enrolled in this study were randomly allocated to training (n=354) and validation (n=88) sets at a ratio of 8:2. No statistically significant differences were observed in baseline demographic, clinical, or laboratory data between the two sets (P>0.05). Univariate logistic regression analysis of the collected data was performed to screen for factors associated with bone loss in patients with RA (**Figure 1**), which identified age, hospitalization stay, course of disease, BMI, ESR, AST/ALT, albumin levels, uric acid levels, RBC, Th1 (%), Th1/Th2, Th1/Treg, total T lymphocyte (%), NK cell count, NK cells (%), and IL-6 levels (P<0.05). Subsequently, variables with statistically significant differences identified by univariate logistic regression were further analyzed using the random forest algorithm, and importance ranking was performed based on the mean decrease Gini index (**Figure 2A**). The optimal number of variables was determined using the OOB error curve. The results showed that the OOB error rate was lowest (approximately 0.20) when the top eight variables by importance ranking were included, indicating optimal discriminative performance of the model (**Figure 2B**). Therefore, these eight variables—age, IL-6 levels, albumin levels, Th1/Treg, disease course, BMI, uric acid levels, and Th1/Th2—were selected for multivariate logistic regression analysis (**Supplementary Table 1**). The results identified age, IL-6 levels, albumin levels, Th1/Treg, BMI, and uric acid levels as factors independently associated with low bone mass in patients with RA (all BH-adjusted P-values <0.05). A personalized nomogram for estimating the probability of concurrent low bone mass in patients with RA was constructed based on these six independent associated factors (**Figure 2C**).

**Figure 1:**
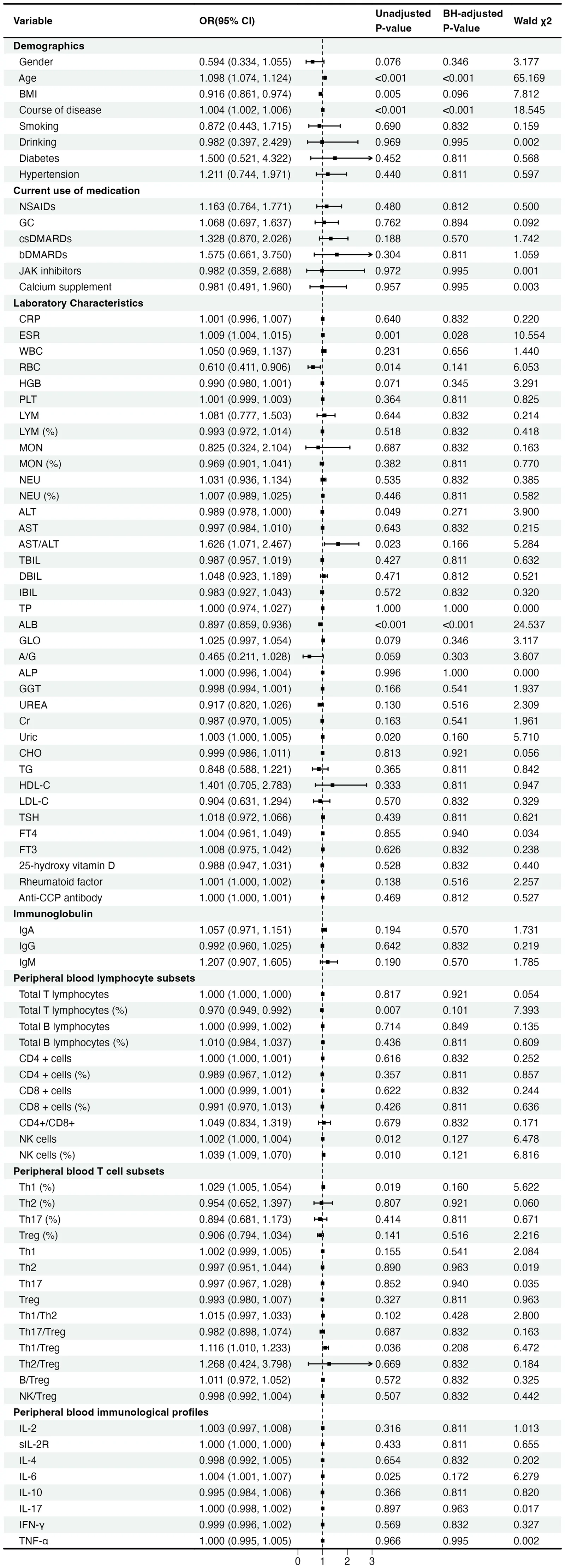
Univariate logistic regression analysis of factors associated with low bone mass in RA patients. OR, odds ratio; 95% CI, 95% confidence interval.

**Figure 2:**
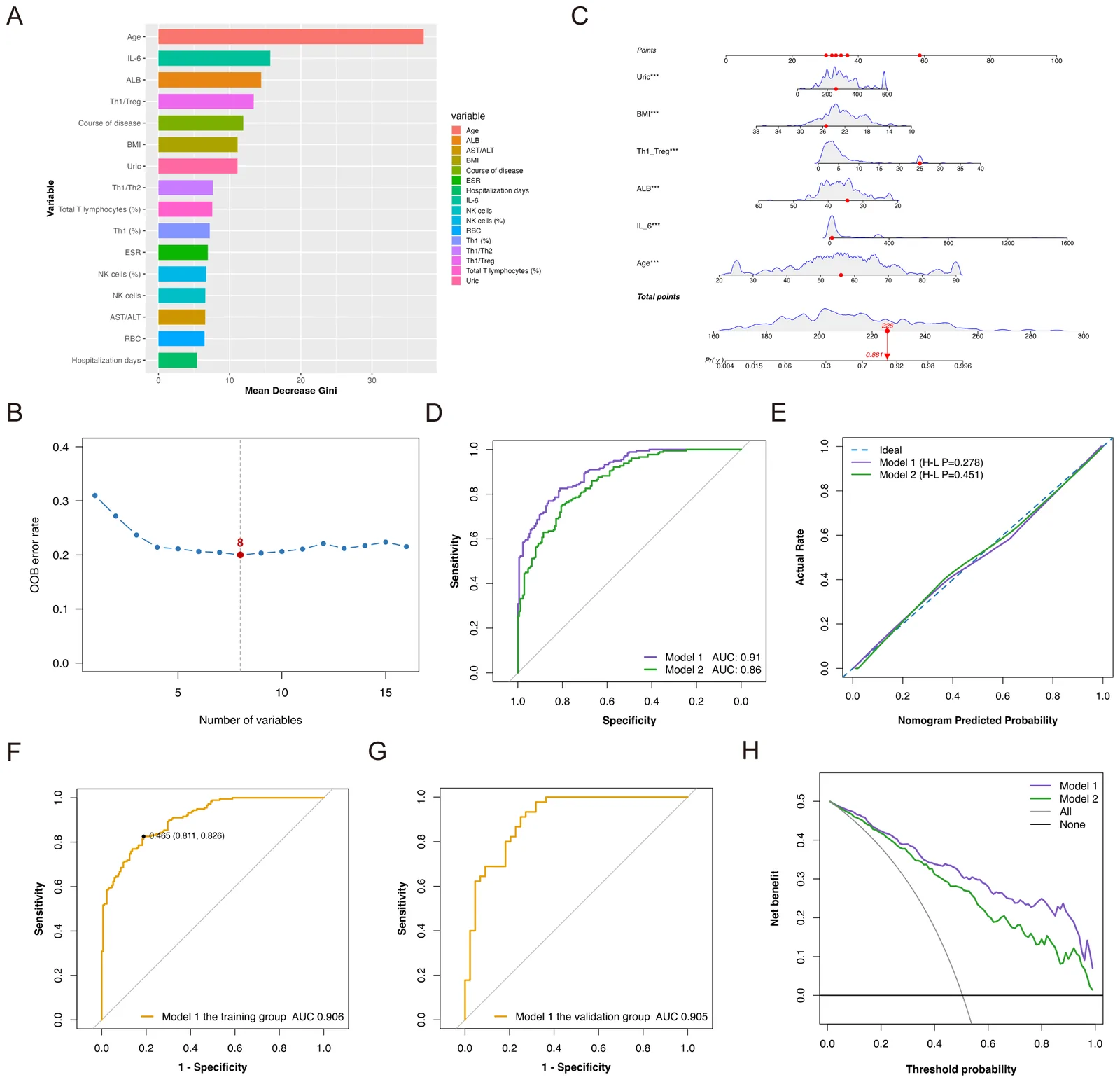
Construction and validation of a nomogram for predicting low bone mass risk in RA patients. **(A)** Importance ranking of factors associated with bone loss in RA patients (Mean Decrease Gini). **(B)** Relationship between the number of variables and OOB error rate for identifying concurrent low bone mass. **(C)** Nomogram for identifying concurrent low bone mass in RA patients. **(D)** ROC curves for identifying concurrent low bone mass in RA patients. Model 1: includes Age, IL-6, ALB, Th1/Treg, BMI, and Uric; Model 2: includes Age, ALB, BMI, and Uric. **(E)** Calibration curves of Model 1 and Model 2. **(F)** ROC curve of Model 1 in the training set. **(G)** ROC curve of Model 1 in the validation set. **(H)** DCA of Model 1 and Model 2.

### 3.5 Validation of the nomogram

IL-6 levels and Th1/Treg cells are not traditional risk factors for bone loss. To determine whether the exclusion of immune-inflammatory indicators would affect the discriminative performance of the model, the nomogram incorporating all six indicators (**Figure 2C**) was designated as model 1, whereas model 2 was constructed to include age, albumin levels, BMI, and uric acid levels. The AUC of model 1 was 0.906 (95% CI: 0.877–0.935), whereas that of model 2 was 0.861 (95% CI: 0.824–0.897) (**Figure 2D**; **Table 4**); this difference between the two models was statistically significant (P = 0.001), indicating that model 1 outperformed model 2. The calibration of the models was assessed using the Hosmer–Lemeshow goodness-of-fit test. The results demonstrated that the calibration curve of model 1 closely approximated the diagonal line, and the Hosmer–Lemeshow test yielded P>0.05, indicating no significant discrepancy between the predicted and observed probabilities (**Figure 2E**). These findings suggest that model 1 possesses a favorable calibration ability, with the predicted values closely approximating the true values. The discriminative abilities of the models were evaluated using receiver operating characteristic (ROC) curve analysis. The results showed that the AUC of model 1 was 0.906 in the training set and 0.905 in the validation set, indicating good discriminative performance (**Figures 2F, G**). For model 2, the corresponding AUCs were 0.861 in both the training and validation sets (**Supplementary Figures 2A, B**). ROC analysis determined the optimal cutoff value of model 1 to be 0.465 (approximately 46.5%) (**Figure 2F**). Decision curve analysis revealed that the model achieved a positive net benefit near this threshold probability (**Figure 2H**). When the threshold probability was set at 46.5%, the net benefit of the model was approximately 0.335. (**Figure 2H**).

**Table 4:** Multivariable logistic regression analysis of factors associated with bone loss in RA patients in models 1 and 2.

Variables	Model 1		Model 2		P
	OR (95% CI)	P	OR (95% CI)	P	
Age	1.137 (1.100-1.175)	<0.001***	1.119 (1.089-1.149)	<0.001***	0.001**
ALB	0.869 (0.818-0.922)	<0.001***	0.871 (0.825-0.920)	<0.001***	
BMI	0.803 (0.728-0.885)	<0.001***	0.829 (0.762-0.901)	<0.001***	
Uric	1.006 (1.003-1.009)	<0.001***	1.004 (1.002-1.007)	<0.001***	
IL-6	1.005 (1.001-1.010)	0.013*	–	–	
Th1/Treg	1.135 (1.060-1.216)	<0.001***	–	–	
AUC	0.906 (0.877-0.935)		0.861 (0.824-0.897)		

OR, odds ratio; 95% CI, 95% confidence interval; AUC, area under the ROC curve. Model 1 includes inflammation markers, Model 2 excludes them. *P<0.05, **P<0.01, ***P<0.001.

## 4 Discussion

RA is a chronic systemic autoimmune disease characterized by chronic synovitis and progressive joint destruction, affecting approximately 0.2–1% of the global population (27). Local and systemic inflammation in RA causes joint mobility limitations and leads to significant bone loss, increasing the risk of fractures and osteoporosis, thereby affecting patients’ quality of life and prognosis (5, 28). Traditional BMD and bone metabolic markers can reflect the extent of bone loss in RA; however, they lack value for predicting the occurrence of bone loss. Traditional factors associated with RA-related bone loss include age, sex, nutritional status, β-C-terminal telopeptide, and disease duration (18, 29). Although previous studies have explored traditional risk factors for bone loss in RA, reliable models for precise prediction and early intervention are lacking.

The pathogenesis of RA involves the activation of multiple immune cells, and the inflammatory state in patients with RA exerts continuous effects on immune cells and other inflammatory components *in vivo* (30, 31). Abnormally activated immune cells and inflammatory factors lead to immune dysregulation, which may be an important mechanism in bone loss (22, 32, 33). Therefore, constructing a nomogram integrating traditional risk factors with immune-inflammatory factors is crucial for the personalized assessment of bone loss risk in patients with RA and for facilitating early intervention.

In our study, univariate and multivariate logistic regression analyses identified advanced age, elevated uric acid levels, decreased BMI, and reduced albumin levels as factors independently associated with low bone mass in patients with RA, which is consistent with previous reports (34, 35). Advancing age is a well-established non-modifiable factor for bone mass loss. With aging, senescence leads to decreased osteoblast activity and enhanced osteoclast activity within bone tissue, resulting in bone resorption exceeding bone formation (36). Elevated uric acid levels are accompanied by the accumulation of oxidative stress substances and increased release of proinflammatory factors, which suppress osteogenesis, promote osteoclastogenesis, and exacerbate bone resorption (37). Although uric acid did not show significant differences after BH correction in univariate analysis, multivariate analysis revealed its independent discriminatory value, suggesting that uric acid participates in the development of bone loss in RA through interactions with other factors. Decreased BMI and albumin levels indicate insufficient nutritional reserves, and malnutrition, sarcopenia, and bone loss represent pathologically continuous stages; malnutrition is closely associated with bone loss in patients with RA (38). These traditional risk factors collectively accelerate the onset and presence of low bone mass under persistent inflammatory conditions in RA. This study also revealed abnormal ESR, GLO, and IgA levels in the RA-LBM group, suggesting significant immune-inflammatory dysregulation in patients with RA with bone loss and the potential role of immune-inflammatory factors in bone loss in RA.

Our study identified IL-6 levels and the Th1/Treg ratio as factors independently associated with low bone mass in RA, suggesting a potential relationship between immune-inflammatory imbalance and altered bone metabolism in patients with RA. In recent years, osteoimmunology research has revealed significant immune dysregulation in patients with RA, including elevated Th17/Treg ratios, excessive B cell activation, and increased levels of proinflammatory cytokines such as IL-6, IL-17, and TNF-α (39). These immune abnormalities drive synovial inflammation, directly activate osteoclasts, and promote bone resorption through the RANKL-OPG axis and non-RANKL-dependent immune complex pathways (13). Multiple studies have associated immune indicators with decreased BMD and increased fracture risk, suggesting that systematic monitoring of immune cell subsets and inflammatory cytokine levels may facilitate the identification of high-risk RA populations at an early stage before significant bone loss occurs, thereby providing an optimal window for targeted bone-protective therapy (22, 40, 41). By incorporating IL-6 and Th1/Treg cells into the model, the present study evaluated their additional discriminatory value beyond conventional factors. Elevated circulating IL-6 levels and an increased Th1/Treg ratio were associated with low bone mass in patients with RA. However, the cross-sectional design does not establish causality or therapeutic relevance. These findings should therefore be considered hypothesis-generating and require prospective and mechanistic validation. Although IL-6 and the Th1/Treg ratio may provide additional immune-inflammatory information, their incremental value over conventional clinical models requires further validation. FRAX estimates future fracture risk rather than concurrent DXA-defined low bone mass and is therefore not directly comparable with our model. In addition, the specialized measurement, standardization, availability, cost, and treatment sensitivity of these immune biomarkers may limit their routine clinical application.

IL-6 is a proinflammatory cytokine that plays a central role in the pathogenesis of RA. IL-6 activates the JAK-STAT signaling pathway, promoting synovial fibroblast proliferation and inflammatory mediator release, thereby leading to joint inflammation and impaired mobility (42). IL-6 enhances osteoclast differentiation and activity by inducing RANKL expression and suppressing osteoprotegerin secretion while concurrently inhibiting osteoblast differentiation and function, resulting in a negative balance in bone remodeling (43, 44). In this study, IL-6 levels were significantly higher in the RA-LBM group than in the RA-N group, and IL-6 was independently associated with low bone mass, suggesting that IL-6 may provide additional information for identifying concurrent low bone mass in patients with RA. This finding is consistent with previous studies demonstrating associations of IL-6 levels with joint erosion and decreased systemic BMD in patients with RA (45).

Treg cells are pivotal in maintaining immune tolerance and suppressing excessive inflammatory responses, whereas Th1 cells mediate cellular immune responses through secretion of cytokines such as IFN-γ. The Th1/Treg ratio reflects the balance between pro- and anti-inflammatory immunity, and an elevated ratio indicates dysregulated immune homeostasis. In RA, reduced Treg cell numbers or functional defects lead to weakened immunosuppressive capacity, whereas excessive Th1 cell activation exacerbates inflammatory responses, highlighting the Th1/Treg imbalance as a critical factor driving autoimmunity and tissue damage (46). This study found that the Th1/Treg ratio was significantly higher in the RA-LBM group than in the RA-N group and was independently associated with low bone mass, suggesting that Th1/Treg imbalance may reflect immune dysregulation associated with concurrent low bone mass in patients with RA. Although an elevated Th1/Treg ratio could theoretically be linked to altered bone metabolism through reduced Treg-mediated immunosuppressive activity and enhanced inflammatory responses, the present findings do not establish such a mechanistic relationship. Further prospective and mechanistic studies are needed to clarify whether Th1/Treg imbalance plays a causal role in RA-related bone loss. In addition, because Th1 cells were quantified after PMA/ionomycin stimulation, these measurements reflect inducible cytokine-producing capacity under ex vivo stimulation rather than unstimulated circulating Th1 activity *in vivo*. Accordingly, the observed Th1/Treg ratio should not be interpreted as a direct measure of the *in vivo* Th1/Treg balance.

To evaluate the incremental discriminatory value of immune-inflammatory indicators, we constructed model 2, which included only traditional risk factors, and compared it with model 1. The results demonstrated that model 1, which incorporated IL-6 and Th1/Treg cells, significantly outperformed model 2, indicating that the inclusion of immune-inflammatory indicators substantially enhanced the discriminative ability of the model. Model 1 showed similar AUCs in the training and validation sets, with favorable calibration findings in the current internal validation. However, given the relatively small validation set and the use of a single random split, these findings should be interpreted cautiously. Decision curve analysis further confirmed that model 1 achieved a net benefit of approximately 0.335 when the threshold probability was set at 46.5%. The nomogram may assist in identifying patients with RA who have a higher probability of concurrent low bone mass. However, clinically meaningful decision thresholds and their corresponding clinical actions require prospective evaluation. Collectively, these results support the clinical value of incorporating immune-inflammatory indicators in the identification of concurrent low bone mass in RA.

Sex and menopausal status are important factors affecting bone health in patients with RA. Previous studies have demonstrated that the prevalence and risk factors for osteoporosis differ among men, premenopausal women, and postmenopausal women with RA, with postmenopausal women showing a particularly high prevalence of osteoporosis (47). Moreover, a recent RA-specific osteoporosis prediction model identified female sex as an independent predictor of osteoporosis (48). These findings highlight the potential influence of sex and menopausal status on bone mass in RA. However, menopausal status was not systematically documented in our retrospective dataset and therefore could not be incorporated into the present analysis. Residual confounding related to menopausal status cannot be excluded, and future prospective studies should systematically collect menopausal information and further evaluate sex- and menopause-specific differences in the determinants of low bone mass in RA.

This study had some limitations. First, the primary outcome was defined as BMD-defined low bone mass (T-score < −1.0) based on available DXA data. Because information on previous fragility fractures was incomplete, clinical osteoporosis could not be reliably classified. Therefore, potential heterogeneity between osteopenia and osteoporosis should be addressed in future studies. Second, glucocorticoid use was analyzed as a dichotomous variable because dose and duration were not uniformly documented and cumulative exposure could not be calculated; this may incompletely account for the known dose-dependent effect of glucocorticoids on bone. Future prospective studies should incorporate cumulative glucocorticoid exposure. Third, BMD assessment may have been influenced by factors such as age, glucocorticoid exposure, or perceived osteoporosis risk. Therefore, the study population may represent hospitalized RA patients selected for BMD assessment rather than the overall hospitalized RA population, potentially introducing selection bias. Fourth, the exclusive inclusion of hospitalized patients with RA may limit the generalizability of our findings, as these patients may have greater disease activity and different immune-inflammatory profiles than stable outpatient populations. External validation in broader RA populations is therefore needed. Fifth, the data-driven variable-selection process may have excluded potentially informative predictors and overestimated model performance. Future studies should validate the model using predefined predictors and independent external cohorts. Sixth, the relatively small validation set (n=88) and the use of a single random split may have affected the stability of the performance estimates. Therefore, the current findings should be regarded as internal validation rather than definitive evidence of model generalizability. Further validation using resampling methods and independent external cohorts is needed. Finally, validated composite measures of RA disease activity, such as DAS28, CDAI, or SDAI, were not systematically available in this retrospective dataset. Given the close relationship between IL-6 and systemic inflammatory activity, residual confounding by overall RA disease activity cannot be excluded. Therefore, whether the association between IL-6 and low bone mass is independent of RA disease activity requires further validation in prospective studies incorporating standardized disease activity measures.

## 5 Conclusion

In this single-center retrospective cross-sectional study of patients with RA, a model incorporating age, BMI, albumin, uric acid, IL-6, and the Th1/Treg ratio showed good discrimination for identifying concurrent BMD-defined low bone mass. Prospective multicenter studies are needed to determine whether these biomarkers improve the prediction of future bone loss or fracture beyond established clinical risk factors.

## Data Availability

The original contributions presented in the study are included in the article/**Supplementary Material**. Further inquiries can be directed to the corresponding authors.
