# Supplementary material for: Development of an IL-6- and Th1/Treg-based nomogram for identifying low bone mass in patients with rheumatoid arthritis
Source: Front Immunol. 2026 Oct 21;17:1952321. doi: 10.3389/fimmu.2026.1952321 (PMC13731378; doi:10.3389/fimmu.2026.1952321)
Supplement: Supplementary file 1 [file DataSheet1.docx]

**Supplementary Figure 1. RA patient screening and grouping flowchart.** BMD, bone mineral density; RA, rheumatoid arthritis; LBM, Low bone mass; N, Normal bone mass.
