# Supplementary material for: Development of an IL-6- and Th1/Treg-based nomogram for identifying low bone mass in patients with rheumatoid arthritis
Source: Front Immunol. 2026 Oct 21;17:1952321. doi: 10.3389/fimmu.2026.1952321 (PMC13731378; doi:10.3389/fimmu.2026.1952321)
Supplement: Supplementary file 2 [file DataSheet2.docx]

**Supplementary Figure 2. ROC analysis of model 2.** (A) ROC curve of model 2 in the training set. (B) ROC curve of model 2 in the validation set. AUC, area under the curve; ROC, receiver operating characteristic.
