# Supplementary material for: Development of an IL-6- and Th1/Treg-based nomogram for identifying low bone mass in patients with rheumatoid arthritis
Source: Front Immunol. 2026 Oct 21;17:1952321. doi: 10.3389/fimmu.2026.1952321 (PMC13731378; doi:10.3389/fimmu.2026.1952321)
Supplement: Supplementary file 3 [file Table1.docx]

| **Variables** | **OR** | **95%CI** | **P** |
| --- | --- | --- | --- |
| Age | 1.133 | 1.097-1.171 | <0.001*** |
| IL-6 | 1.005 | 1.001-1.009 | 0.019* |
| ALB | 0.867 | 0.817-0.921 | <0.001*** |
| Th1/Treg | 1.131 | 1.060-1.207 | <0.001*** |
| Course of disease | 1.002 | 0.999-1.005 | 0.135 |
| BMI | 0.801 | 0.725-0.884 | <0.001*** |
| Uric | 1.006 | 1.003-1.009 | <0.001*** |
| Th1/Th2 | 1.010 | 0.988-1.032 | 0.385 |

Supplementary Table 1. Multi-factor logical regression analysis of factors related to bone loss in AS patients
